# Biochemical and Mutational Analysis of a Novel Nicotinamidase from *Oceanobacillus iheyensis* HTE831

**DOI:** 10.1371/journal.pone.0056727

**Published:** 2013-02-25

**Authors:** Guiomar Sánchez-Carrón, María Inmaculada García-García, Rubén Zapata-Pérez, Hideto Takami, Francisco García-Carmona, Álvaro Sánchez-Ferrer

**Affiliations:** 1 Department of Biochemistry and Molecular Biology-A, Faculty of Biology, Regional Campus of International Excellence “Campus Mare Nostrum”, University of Murcia, Campus Espinardo, Murcia, Spain; 2 Murcia Biomedical Research Institute (IMIB), Murcia, Spain; 3 Microbial Genome Research Group, Institute of Biogeosciences, Japan Agency for Marine-Earth Science and Technology (JAMSTEC), Yokosuka, Kanagawa, Japan; Laurentian University, Canada

## Abstract

Nicotinamidases catalyze the hydrolysis of nicotinamide to nicotinic acid and ammonia, an important reaction in the NAD^+^ salvage pathway. This paper reports a new nicotinamidase from the deep-sea extremely halotolerant and alkaliphilic *Oceanobacillus iheyensis* HTE831 (OiNIC). The enzyme was active towards nicotinamide and several analogues, including the prodrug pyrazinamide. The enzyme was more nicotinamidase (*k_cat_/K_m_* = 43.5 mM^−1^s^−1^) than pyrazinamidase (*k_cat_/K_m_* = 3.2 mM^−1^s^−1^). Mutational analysis was carried out on seven critical amino acids, confirming for the first time the importance of Cys133 and Phe68 residues for increasing pyrazinamidase activity 2.9- and 2.5-fold, respectively. In addition, the change in the fourth residue involved in the ion metal binding (Glu65) was detrimental to pyrazinamidase activity, decreasing it 6-fold. This residue was also involved in a new distinct structural motif **D**A**H**XXXDXXHP**E** described in this paper for Firmicutes nicotinamidases. Phylogenetic analysis revealed that OiNIC is the first nicotinamidase described for the order Bacillales.

## Introduction

Nicotinamidases (EC 3.5.1.19) catalyze the deamination of nicotinamide (NAM) to produce ammonia and nicotinic acid (NA) (Fig. S1 in the supplemental material, shadowed area). *In vivo,* the latter compound is converted back to NAD^+^ in a series of reactions catalyzed by other enzymes in the NAD^+^ salvage pathways. Nicotinamidases are key enzymes in many organisms, including bacteria [1], mycobacteria [2], [3], yeasts [4], [5], [6], [7], protozoa [8], [9], plants [10] and even in invertebrates, such as *Drosophila melanogaster* [11] or *Caenorhabditis elegans* [12]. However, mammalian genomes do not encode nicotinamidases, and use nicotinamide phosphoribosyltransferase to convert NAM to nicotinamide mononucleotide (NMN), which is later adenylated back to NAD^+^. Mammals are also capable of using nicotinic acid to make NAD^+^ via the Preiss-Handler pathway [13]. Recent studies have indicated the importance of nicotinamidase activity for the viability and proliferation of organisms that are pathogenic to humans, such as *Borrelia burgdorferi* (involved in Lyme disease) [14], [15] and *Brucella abortus* (involved in Malta fever) [16]. In addition, the increased nicotinamidase activity detected in erythrocytes infected with *Plasmodium falciparum* [8] suggests that this pathogenic organism requires the enzyme as well, since it does not appear to encode genes for the enzymes involved in *de novo* NAD^+^ synthesis [1], [17]. Moreover, the *Leishmania infantum* nicotinamidase (LiPnc1) is essential for NAD^+^ production and parasite proliferation [9], [18]. The above facts, together with the absence of nicotinamidase in human NAD^+^ biosynthetic pathways, have increased interest in this enzyme as a possible drug target, suggesting that small molecule inhibitors of nicotinamidases could serve as specific agents for the above mentioned pathogenic organisms [1], [17].

In addition, NAM is the substrate of two distinct enzymes, nicotinamidases and nicotinamide phosphoribosyltransferases (Nampt) (only found in higher vertebrates), with equivalent function in the NAD^+^ salvage pathways. This compound, NAM, is both the product of [19], [20] and a negative feed-back inhibitor of NAD^+^ consumers [19], [21], [22], including PARPs and sirtuins. These last NAD^+^-dependent deacetylases are widely distributed in biology and play a crucial role in several cellular processes, such as gene silencing, elongation of the life-span, metabolism and chromatin structure [23]. Thus, nicotinamidases have received considerable attention as prolongers of lifespan in various organisms such as *D. melanogaster* [11] and *Saccharomyces cerevisiae* [24]–[26], through their depletion of intracellular NAM concentrations, thereby increasing sirtuin activity. However, this is not the only mechanism to regulate sirtuins, since perturbation of the salvage pathway at points that do not perturb NAM levels and alterations in the NAD^+^/NADH ratio, as a result of enhanced NAD^+^ synthesis, also have an impact on their activity [27]–[30].

The above described important biological effects of sirtuins (recently reviewed in references [31]–[33]) have intensified the search for new modulators (activators and inhibitors) of sirtuins, in which nicotinamidases will undoubtedly play another crucial role as a key enzyme in the continuous high-throughput spectrophotometric assay, which couples the activity of sirtuins with nicotinamidase and glutamate dehydrogenase [34] (Fig. S1). Although this last enzyme can be purchased from different commercial sources, nicotinamidase is not commercially available. Thus, an efficient nicotinamidase overexpression and purification method could be of great biotechnological interest for the screening of new sirtuin modulators.

These enzymes are usually classified in the databases into three classes depending on their ability to convert more efficiently nicotinamide (NAM) and/or pyrazinamide (PZA): nicotinamidases, such as those of *S. cerevisiae* Pnc1 (5) or *Streptococcus pneumoniae* SpNIC (1); bifunctional pyrazinamidases/nicotinamidases, such as those of *Pyrococcus horikoshii* PhPncA [35] and *Acinetobacter baumanii* AbPncA [36]; or pyrazinamidases, such as the one of *M. tuberculosis* MtPncA [37]. Together with rifampicin and isoniazid [36], PZA is an important front-line tuberculosis pharmaceutical, and mutations in this enzyme are usually associated with resistance to PZA [7].

The aim of this paper was to characterize a new nicotinamidase from the deep-sea extremely halotolerant and alkaliphilic *Oceanobacillus iheyensis* HTE831, isolated from a depth of 1050 m on the Iheya Ridge [38]. The enzyme (OiNIC) was not only active towards nicotinamide but also towards a wide range of nicotinamide analogues, including the pro-drug pyrazinamide. OiNIC was found to be a good catalyst (*k_cat_* of 11.6 s^−1^ for NAM and 2.6 s^−1^ for PZA) and stable from acid to neutral pH values. Several mutants were designed for the first time in an attempt to increase the pyrazinamidase activity of OiNIC, and C133A and F68W were seen to improve the catalytic efficiency towards this pro-drug. Finally, a study of the distribution of nicotinamidases across biology and a phylogenetic analysis of bacterial nicotinamidases were carried out for the first time in order to deepen our understanding of the evolution of these enzymes. Among other findings, OiNIC is the first nicotinamidase to be described for the order of Bacillales.

## Materials and Methods

### Strains, Plasmids, and Chemicals

Genomic DNA was isolated from *Oceanobacillus iheyensis* HTE831 deposited in JAMSTEC (Japan) [39]. The pTYB21 vector was from New England Biolabs. The pET28a cloning vector was from Novagen (EMD Bioscience Inc. Madison, WI, USA). QIAquick PCR purification kit and QIAprep spin miniprep kit were from Qiagen (Valencia, CA, USA). *Pfu* DNA polymerase was from Stratagene (La Jolla, CA, USA). NADPH was from Carbosynth (Berkshire, UK), 5-methylnicotinamide was from Alfa Aesar (MA, USA). Other reagents were from Sigma.

### Cloning of the OiNIC Gene

The cloning and transformation techniques used were essentially those described by Sambrook et al. [40]. Genomic DNA from *Oceanobacillus iheyensis* HTE831 was used as the source of nicotinamidase gen (Uniprot code: Q8ESQ6). The 552 bp gene was amplified by PCR using forward primer 5′CGCGGC*CATATG*AAAAAAAAGGCATTATTAAATATCGATTATA-3′ and reverse primer 5′-CGCC*GAATTC*CTATCTTACCTCTGC ACCAAT-3′ (restriction enzyme cleavage sites are italicized). The resulting PCR product was purified and digested with *NdeI* and *EcoRI* restriction enzymes, ligated to the digested Intein-tag pTYB21, which carries a chitin binding domain, and transformed into competent *E. coli* Rosetta 2 (DE3) competent cells (Novagen). A selected clone harboring the correct sequence was denoted as pTYB21-OiNic. *OiNic* gene was also cloned into pET28a vector using the same primers and competent *E. coli* strains in order to attain the high-yield overexpression of the protein. The recombinant vector was called pET28a-OiNic.

### Expression and Purification

The above *E. coli* cells harboring the recombinant plasmid pTYB21-OiNic were cultured in 1 L of Terrific Broth (TB) supplemented with antibiotics and induced by adding 0.4 mM isopropyl-β-D-thiogalactoside (IPTG) for 12 hours at 20°C with constant stirring. The culture was diafiltered through a 500-kDa membrane (GE Healthcare, Uppsala, Sweden) and cleaned with 50 mM Tris-HCl buffer pH 8.0 containing 1 mM EDTA and 25% sucrose. pTYB21-OiNic enzyme was expressed in the form of insoluble inclusion bodies, which is why cells were disrupted by sonication on ice and the cell debris was washed several times with the following inclusion body buffers: 20 mM Tris-HCl pH 8.0 with 0.2 M NaCl, 1% sodium deoxycholate and 2 mM EGTA; and 10 mM Tris-HCl pH 8.0 with 0.25% sodium deoxycholate and 1 mM EGTA. The purification was performed in two steps. Despite the absence of a poly-histidine tag in the recombinant protein, the resulting supernatant was purified by Ni^2+^-chelating affinity chromatography (ÄKTA Prime Plus, GE Healthcare) on a HisTrap Phenyl FF column (GE Healthcare, Uppsala, Sweden) due to the presence of exposed histidine residues in the sequence of the protein that efficiently binds to the Ni^2+^-chelating column. Activity fractions were further purified using a chitin column (New England Biolab) and intein self-splicing was induced with 50 mM DTT to elute the protein. Fractions were desalted, concentrated and stored at −20°C with 10% glycerol and 2 mM DTT.

*E. coli* cells harboring the recombinant plasmid pET28a-OiNic were grown and IPTG-induced (0.5 mM) in 1 L of TB supplemented with antibiotics at 30°C for 12 hours. OiNIC was expressed in the soluble fraction after disruption. The purification was performed in two steps, starting with tangential ultrafiltration with a 50-kDa cut-off membrane on a QuixStand system (GE Lifesciences) followed by Ni^2+^-chelating affinity chromatography (ÄKTA Prime Plus, GE Lifesciences) onto a HiPrep IMAC 16/10 FF 20 mL column (GE Lifesciences). The bound enzyme was eluted with a linear imidazol gradient (0–250 mM). The fractions containing the desired activity were pooled, desalted, concentrated and stored at −80°C.

Gel filtration (Superdex 200, GE Lifesciences) was used to confirm the homogeneity and the molecular mass of the purified enzyme. Superdex-200-purified OiNIC was used for the protein melting experiments and ICP-OES assays. In addition, the molecular mass was determined using an HPLC/ESI/ion trap system [41] and the quaternary structure of the enzyme was confirmed by cross-linking experiments with 3 mg/mL of dimethyl suberimidate (DMS) [42]. The protein concentration was determined using Bradfords reagent (Bio-Rad) and BSA as standard.

### Enzyme Assay

Nicotinamidase cleavage was determined both spectrophotometrically and chromatographically (HPLC). The nicotinamidase spectrophotometric method measured the decrease in absorbance at 360 nm (ε_360nm_ = 4320 M^−1^ cm^−1^) corresponding to the oxidation of NADPH produced by glutamate dehydrogenase (GDH) in the presence of α-ketoglutarate, when NH_3_ appeared as a consequence of the hydrolysis of nicotinamide by OiNIC [34] (Fig. S1). Absorbance was measured at 360 nm instead of 340 nm due to the amount of NAD(P)H used to saturate glutamate dehydrogenase. The standard reaction medium (1 mL) for the above assay, which was carried out in a UV-2401 PC spectrophotometer (Shimadzu), contained 300 µM NADPH, 9.7 µg GDH, 1 mM NAM, 10 mM α-ketoglutarate and 1.3 µg of purified OiNIC in 100 mM phosphate buffer pH 7.3. A control assay without NAM was also carried out to determine the presence of any other NADPH-consuming enzymes. One unit of activity is defined as the amount of enzyme consuming 1 µmol of NADPH in 1 min at pH 7.3 and 37°C. This method was also used to measure NAM analogues, such as pyrazimamide (PZA) and 5-methylnicotinamide. The data refer to three repeated experiments.

The hydrolytic activity was also measured from the increase in the nicotinic acid area, using HPLC (Agilent 1100 series) with a reverse-phase C-18 250×4.6 mm column (Gemini C18, Phenomenex) and a mobile phase (20 mM ammonium acetate pH 6.9) running at 1 mL/min. Under these conditions, the retention time (R_T_) for NAM and NA were 19.9 and 7 min, respectively. One unit of activity was defined as the amount of enzyme required to cleave 1 µmol of NAM releasing 1 µmol of NA in 1 min (HPLC). The standard reaction medium for the HPLC reaction consisted of 1 mM NAM and 0.67 µg purified OiNIC in 100 mM phosphate buffer pH 7.3. Reactions were stopped by the addition of trifluoroacetic acid to reach a final pH of 3.0. the data refer to three repeated experiments.

### Stability Assays

pH-stability was assayed by spectrophotometrically measuring the residual activity of OiNIC incubated at different pHs at 37°C. A heat-stability assay was carried out by incubating the enzyme at pH 7.3 from 5 to 55°C, using a water bath. Aliquots (50 µL) were taken at different times and cooled on ice before they were spectrophotometrically measured in the standard reaction media.

Melting curves to determine protein unfolding were obtained with Sypro Orange fluorescent dye (Molecular Probes) as described [41]. The *T_m_* values obtained with this method correlate well with those obtained by other biophysical methods such as CD or DSC [43]. The same technique was also used to determine the effect of different additives and buffers on OiNIC melting temperature.

### Kinetic Analysis of Inhibitors

Nicotinaldehydes were characterized as inhibitors, using nicotinamide as substrate. Reactions were performed using the GDH-coupled assay described above, after confirming that these compounds did not inhibit GDH activity. Inhibition reactions contained 10 mM α-ketoglutarate, 300 µM NADPH, 1 mM NAM, 9.7 µg GDH, 1.3 µg of purified OiNIC and varying concentrations of inhibitors in 100 mM phosphate buffer pH 7.3. Rates were fitted to Morrisońs quadratic equation [44], which accounts for the tight binding observed, since all inhibitors tested were found to have an intrinsic *K_i_* of <5 µM:
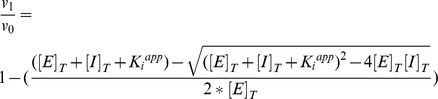
where ν_i_ is the inhibited rate for a given concentration of inhibitor, ν_0_ is the uninhibited rate, [*E*]

n_T_ is the total enzyme concentration, [*I*]_T_ is the total inhibitor concentration, and *K_i_^app^* is the apparent inhibition constant. The intrinsic binding constant for binding of the inhibitor to the enzyme, *K_i_*, can be calculated from *K_i_^app^* by the equation:
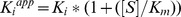
where [S] is the substrate concentration and *K_m_* is the Michaelis constant for binding of the substrate to the enzyme, both in the same units as [*I*]_T_.

### Site Directed Mutagenesis of OiNIC

Seven single mutants (T12Q, Q96K, Q96A, K104A, C133A, F68W, E65H) and one double-mutant (C133A/F68W) were constructed using overlap extension PCR [45]. The primers used for amplification are listed in Table S1. pET28a-OiNic doubled stranded plasmid DNA was used as the template. PCR products were digested with *Dpn*I to ensure complete removal of the parental plasmid, and transformed in *E. coli* DH5α electrocompetent cells. All mutations were confirmed by sequencing. The kinetic parameters of mutants were determined as previously described for parental OiNIC.

### Determination of Metal Ion Content

The metal ion content (Fe^2+^, Zn^2+^ and Mn^2+^) of OiNIC was determined by triplicate runs using Inductively Coupled Plasma-Optical Emission Spectrometry (ICP-OES) equipment (Optima 2000 DV, Perkin-Elmer, MA, USA) [3], [36]. Purified OiNIC (1 mL) was diluted to a final concentration of 5.1 mg/mL with 1 mL of HNO_3_ (60%) and digested for 4 hours at 85°C as previously described [37]. A range of calibration standards was prepared using single element 100 mg/L stock solutions, diluted with a mixture containing 30% HNO_3_ and 30 mM Tris-HCl buffer pH 7.3 at four different concentrations: 0.1, 1, 10 and 25 mg/L. The metal ion content of the protein was calculated using the calibration curved obtained for each metal ion after subtracting the background signal in the blank buffer-HNO_3_ mixture.

### In Silico Analysis

BLAST searches were used to identify homologues of nicotinamidases [46]. The sequences were aligned using ClustalW [47] and ESPript [48]. Protein sequences were 3D modelled with Geno 3D [49] and ModWeb [50]. Distribution analysis of nicotinamidases was carried out using the HMMER web server [51] and Uniprot database [52]. A sequence significance E-value threshold of 1e^−45^ (Hit: 3e^−45^) was chosen, in order to eliminate false non-homologous results. Phylogenetic trees were obtained using MEGA 5.0 [53].

## Results

### Amino Acid Sequence Comparison

The deduced amino acid sequence of the *O. iheyensis* nicotinamidase (OiNIC) showed significant identity with those of other species in the database. Sequence alignment indicated that OiNIC had an elevated sequence identity with isochorismatase hydrolases, a subfamily within the cystein-hydrolases superfamily, which also encloses the nicotinamidases/pyrazinamidases. OiNIC showed 53% sequence identity with the crystallized nicotinamidase from the Firmicute *Streptococcus pneumoniae* (PDB codes: 3O90, 3O91, 3O92, 3O93, 3O94) [54], but less sequence identity with other crystallized nicotinamidases, such as those from the Gamma-Proteobacteria *Acinetobacter baumanii* (32%, PDB code: 2WT9, 2WTA) [36], the Actinobacteria *Mycobacterium tuberculosis* (34%, PDB code: 3PL1) [37], the Archaea *Pyrococcus horikoshii* (31%, PDB codes: 1ILW, 1IMS) [35], the yeast *Sacharomyces cerevisiae* (25%, PDB code: 2H0R) [5] and the recently crystallized eukaryotic nicotinamidase from *Leishmania infantum* (31%, PDB code: 3R2J) [9].

**Figure 1:**
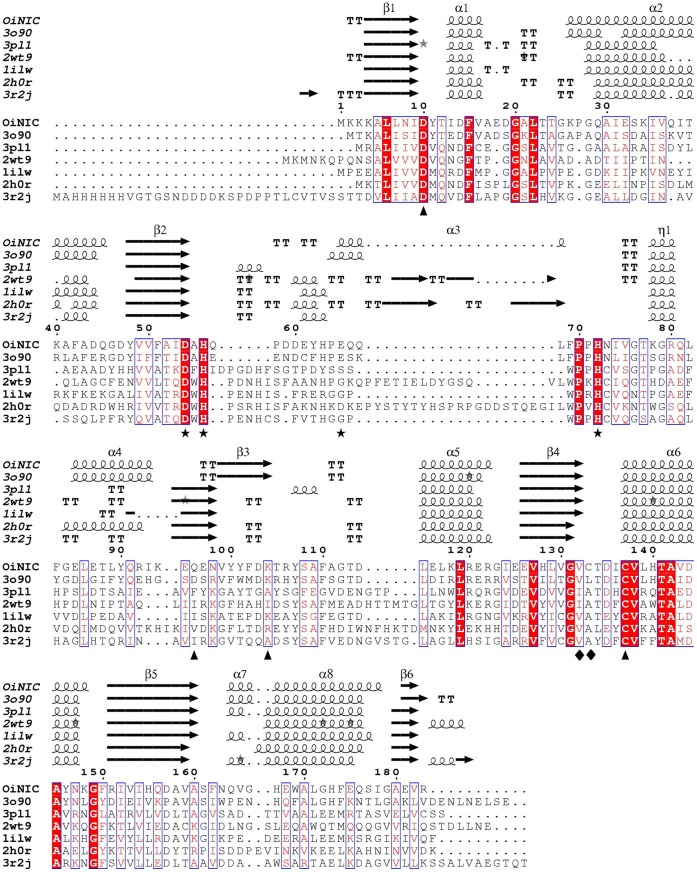
Multiple sequence alignment for *O. iheyensis* nicotinamidase (OiNIC) and related nicotinamidases. ESPript outputs [48] obtained with the crystallized nicotinamidase sequences retrieved from Uniprot database and later aligned with CLUSTAL-W [47]. Sequences are grouped according to similarity. PDB codes were 3O90 for *S. pneumoniae* nicotinamidase (SpNIC), 1ILW for *P. horikoshii* nicotinamidase (PhPncA), 3PL1 for *M. tuberculosis* nicotinamidase (MtPncA), 2WT9 for *A. baumanii* nicotinamidase (AbPncA), 2H0R for *S. cerevisiae* nicotinamidase and 2R3J for *Leishmania infantum* nicotinamidase. Residues strictly conserved across nicotinamidase enzymes have a dark background. Symbols above blocks of sequences represent the secondary structure, springs represent helices and arrows represent β-strands. The residues forming the active site are indicated by triangles. Residues involved in the coordination of the metal ion are indicated by stars and residues forming the *cis*-peptide bond are indicated by diamonds.

In addition, sequence alignment revealed that OiNIC contained conserved residues forming the characteristic catalytic triad of the cystein-hydrolases family (Fig. 1, triangles), a catalytic cysteine at position 137 (C137, OiNIC numbering), an aspartate at position 10 (D10), and a lysine at position 104 (K104) [1], [3], [5], [35]. Another conserved feature of the active center is the presence of a *cis*-peptide bond (Fig. 1, diamonds), whose sequence differs between species (Fig. 1, positions V132 and C133; see also Table S2), but which is invariably preceded by a conserved glycine (G131) [35]. Notably, the second residue implicated in the formation of the *cis*-peptide bond in pyrazinamidases, such as that of *Mycobacterium tuberculosis* (MtPncA; PDB: 3PL1), is generally an alanine (Fig. 1, position 133, A134 in MtPncA), which orientates the amide nitrogen atom of the latter A134 towards the active site center, so that it forms a potential oxyanion hole with the nitrogen of C138 [37]. However, in OiNIC and in other Firmicutes (data not shown), it is a cysteine (Fig. 1, position 133) [1]. Finally, the conserved specific metal ion binding motif usually includes one aspartate and two histidines, such as D54, H56, and H72 (Fig. 1, stars). Depending on the metal ion and on the structural conformation of the protein, a fourth residue may be implicated in the metal ion binding, which remains unclear [3], [35], [37]. Structure alignment of OiNIC with *S. pneumoniae* nicotinamidase (SpNIC) and other nicotinamidases (Table S2) suggests that this fourth residue could be glutamate (E65) in nicotinamidases or serine/histidine in pyrazinamidases [54].

Other residues also involved in the formation of the hydrophobic cavity, where nicotinamide and the ion metal bind [35] may also be found in OiNIC, such as T12, F15, D14, L22, F68, Y107, S108 and T141 (Table S2). These residues delimiting the active site are usually conserved among species, except for F68 and T12, which are conserved in nicotinamidases from phylum Firmicutes, such as SpNIC and OiNIC, but not in the crystallized pyrazinamidases, where they are tryptophan and glutamine, respectively (Table S2). This suggests that these residues could be involved in the substrate specificity of nicotinamidases, becoming more or less active towards the pro-drug pyrazinamide.

### Cloning, Overexpression and Purification of OiNIC

The gene encoding the nicotinaminidase enzyme from *Oceanobacillus iheyensis* HTE831 was cloned into pTYB21 vector. The DNA sequence of the cloned gene showed no mutations compared with the *OiNic* gene sequence reported (Uniprot code: Q8ESQ6). The recombinant clone with the highest expression was induced and purified from *E. coli* cells as described in Materials and Methods. After these steps, the enzyme was pure, as shown in SDS-PAGE (Fig. S2, lane 1). The molecular mass of purified protein was determined by gel filtration (40.9 kDa) and by HPLC/ESI/ion trap (21.1 kDa), confirming the dimeric nature of OiNIC. To further confirm this dimeric conformation of OiNIC, a cross-linking experiment with dimethyl suberimidate (DMS) was carried out. After 8 hours incubation with DMS at room temperature, OiNIC dimer (42 kDa) also became evident in SDS-PAGE (Fig. S2, lane 2).

### Biochemical Characterization of Recombinant OiNIC

**Figure 2:**
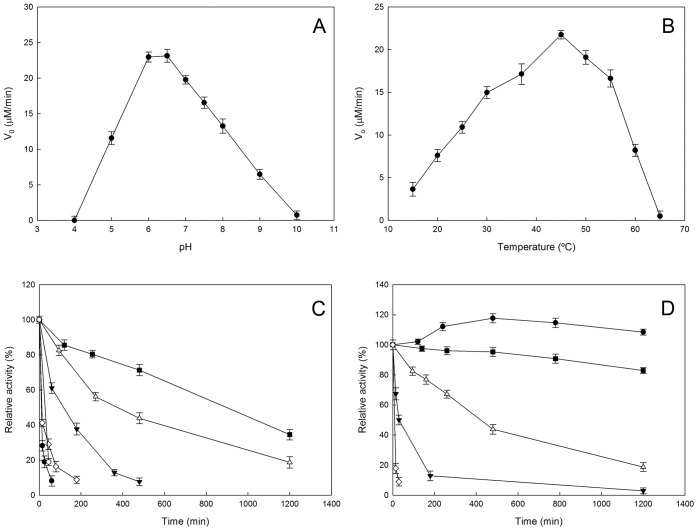
Effect of pH and temperature on OiNIC activity and stability. A) pH profile for OiNIC determined by HPLC. The assay conditions at 37°C were 1 mM nicotinamide, 0.67 µg of OiNIC. The buffers (100 mM) used were sodium acetate pH 4.0–6.0, sodium phosphate pH 7.0–8.0, Tris-HCl pH 9.0 and glycine pH 10.0. B) Temperature profile. Assay conditions were the same as above at pH 7.3 at different temperatures from 15 to 65°C. C) pH stability. OiNIC was incubated at 37°C at pH 5 (•), pH 6 (▪), pH 7.3 (Δ), pH 8 (▾), pH 9 (◊) and pH 10(○). Buffer compositions were the same as above. Residual activity was measured spectrophotometrically under the standard reaction at 37°C. D) Temperature stability. OiNIC was incubated at 4 (•), 20 (▪), 37 (Δ), 45 (▾) and 55°C (◊) in 100 mM sodium phosphate buffer pH 7.3. Residual activity was measured spectrophotometrically using the standard reaction medium.

The enzyme activity was both pH- and temperature-dependent (Fig. 2). Optimum pH and temperature could not be measured by the spectrophotometric assay due to the distortion caused by the coupling enzyme glutamate dehydrogenase (GDH). To solve this, HPLC was used to measure these data (see Materials and Methods). The optimal pH of OiNIC was found to be around pH 6.0–6.5, with a marked decrease in activity below pH 5.0 and above pH 8.0 (Fig. 2A). OiNIC enzyme exhibited its maximum activity at a temperature close to 45°C. Below 25°C or above 55°C, the enzyme lost most of its activity (Fig. 2B). These results are consistent with the few data in the bibliography, where the optimum temperatures reported range from 30 to 40°C [3], [55], [56]. However, and comparison purposes [1], [3], stability and kinetic experiments were carried out at pH 7.3 and 37°C.

**Figure 3:**
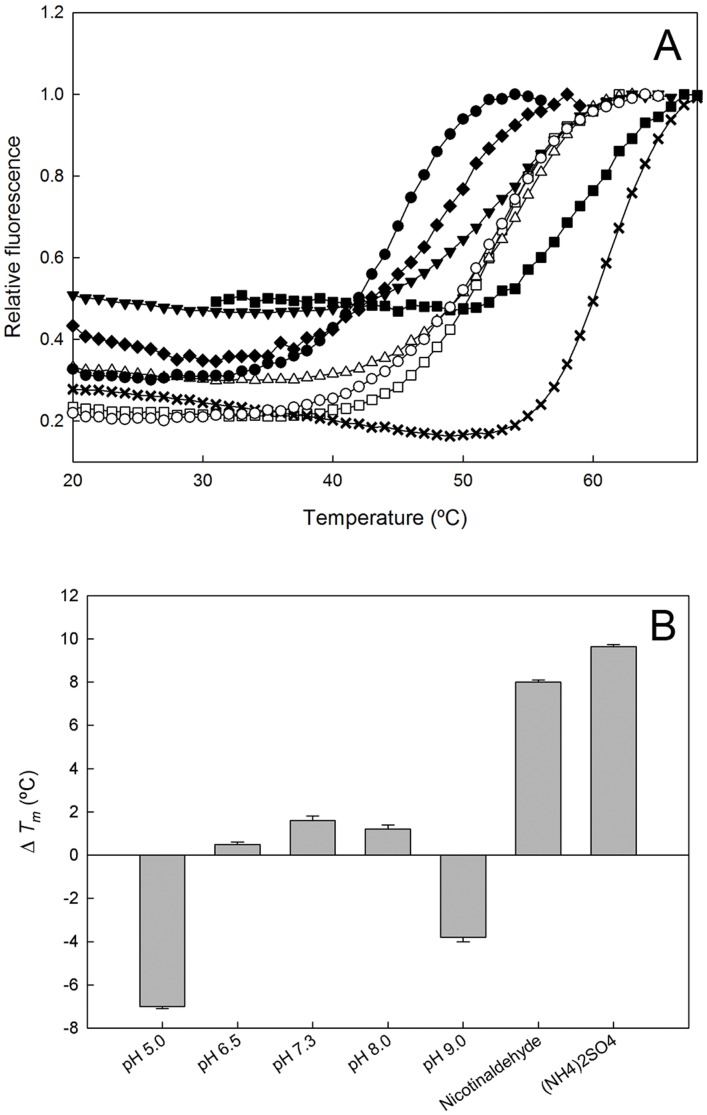
Thermal stability of OiNIC. A) Melting temperature curves of purified enzyme (1 µg) were obtained in the presence of fluorescent probe SYPRO Orange in Milli Q water (○), 100 mM sodium acetate pH 5.0 (•), 100 mM sodium phosphate pH 6.5 (□), pH 7.3 (Δ), pH 8.0 (▾), 100 mM TRIS-HCl pH 9 (♦), 1 mM nicotinaldehyde (▪) and 1 M ammonium sulfate (x) prepared in 100 mM sodium phosphate pH 7.3. B) Effect of different modulators on the melting temperature of OiNIC. Differences in Δ*T_m_* were calculated subtracting MilliQ® *T_m_* value from the *T_m_* values obtained for the enzyme under the different conditions used above.

Interestingly, OiNIC was very stable at pH 6.0 and 7.3, where it maintained, after 20 hours of incubation, 40% and 30% residual activity, respectively (Fig. 2C). The thermostability of OiNIC was studied both spectrophotometrically by incubating the enzyme at different temperatures and by thermal shift assays (TSA) as described in Materials and Methods. OiNIC was seen to be stable at 4°C and 20°C for 20 hours (Fig. 2D). Strikingly, a slight increase in relative activity to over 100% was observed when the enzyme was incubated at 4°C (Fig. 2D, circles). The enzyme maintained 50% activity after 1 hour at 45°C (Fig. 2D, filled triangles), but dropped quickly when incubated at 55°C (Figure 2D, diamonds). These data were also similar to those found when TSA was carried out in MilliQ® water (Fig. 3A, semi-filled diamonds) and in 100 mM buffered solutions at different pHs (Fig. 3A). *T_m_* was ∼51.7±0.2°C in MilliQ® water, increasing by about 2°C in buffered solutions at pHs 7.3 and 8.0. (53.3±0.2°C and 52.9±0.2°C, respectively), but falling by 4°C above pH 9.0 (*T_m_* 47.9±0.2°C). At pH 6.5, *T_m_* was similar to that of MilliQ® water (52.2±0.1°C), which was 9°C higher than the *T_m_* described for *M. tuberculosis* pyrazinamidase at pH 6.0 [37]. A more acidic pH (pH 5.0) lowered *T_m_* drastically to 44.7±0.3°C. TSA was also used to study the binding of different molecules to the OiNIC structure (Fig. 3B). The addition of a competitive inhibitor, nicotinaldehyde, to the enzyme produced an increase in *T_m_* of about 8°C (*T_m_:* 59.7±0.3°C), which was similar to that found with a known protein stabilizer, such as ammonium sulfate (61.3±0.2°C).

### Substrate Specificity and Kinetic Parameters

The specific activity of OiNIC was studied towards nicotinamide and some of its derivatives (Table 1), which include pyrazinamide, 5-methylnicotinamide and two nicotinate esters (methylnicotinate and ethylnicotinate). The enzyme showed a clear preference for nicotinamide rather than pyrazinamide (23.3±0.5 *vs* 3.6±0.5 U/mg). This was also reflected in its kinetic parameters (Table 1). The *K_m_* values were 0.26±0.02 mM for NAM and 0.81±0.01 mM for PZA. The catalytic efficiency of OiNIC towards NAM was 43.48 mM^−1^ s^−1^, whereas for PZA it was only 3.20 mM^−1^ s^−1^, about 13-fold lower.

**Table 1:** Kinetic parameters of wild-type OiNIC and its mutants.

	Specific activity (U/mg)	*K_m_* (mM)	*k*_cat_ (s^−1^)	*k*_cat_/*K_m_* (mM^−1^s^−1^)
**Wild-type**				
NAM^b^	23.3^a^/25.0 b±0.5	0.26±0.02	11.65±0.1	43.48
PZA^a^	3.62±0.5	0.81±0.01	2.60±0.1	3.20
5-methyl-NAM^a^	9.01±0.1	0.68±0.02	5.10±0.02	7.50
Methylnicotinate^b^	0.45±0.02	1.03±0.01	0.32±0.02	0.31
Ethylnicotinate^b^	0.13±0.01	1.02±0.01	0.10±0.01	0.10
**T12Q**				
NAM^a^	0.37±0.02	N.D.	N.D.	N.D.
**Q96K**				
NAM^b^	8.70±0.2	0.57±0.02	2.37±0.1	4.15
PZA^a^	0.46±0.02	1.15±0.05	0.45±0.03	0.39
5-methyl-NAM^a^	3.96±0.12	0.62±0.01	2.16±0.05	3.40
Methylnicotinate^b^	0.31±0.02	0.44±0.02	0.17±0.02	0.38
Ethylnicotinate^b^	0.06±0.02	1.11±0.03	0.05±0.01	0.05
**Q96A**				
NAM^b^	24.8±0.1	0.36±0.01	16.05±0.3	44.59
PZA^a^	1.80±0.2	1.20±0.03	1.61±0.1	1.34
5-methyl-NAM^a^	28.0±0.1	0.55±0.01	15.90±0.2	29.04
Methylnicotinate^b^	1.16±0.03	0.56±0.01	0.60±0.05	1.17
Ethylnicotinate^b^	0.22±0.02	1.43±0.02	0.19±0.01	0.13
**K104A**				
NAM^a^	0.36±0.01	N.D.	N.D.	N.D.
**C133A**				
NAM^b^	17.50±0.1	0.40±0.01	13.60±0.3	34
PZA^a^	6.39±0.2	0.36±0.02	3.31±0.1	9.19
5-methyl-NAM^a^	11.74±0.2	0.64±0.01	7.09±0.2	11.07
Methylnicotinate^b^	1.40±0.03	0.89±0.03	1.02±0.06	1.14
Ethylnicotinate^b^	0.34±0.02	0.34±0.01	0.14±0.01	0.41
**F68W**				
NAM^a^	19.97±0.2	0.17±0.01	8.79±0.2	52.01
PZA^a^	7.50±0.3	0.50±0.02	3.94±0.1	7.9
**E65H**				
NAM^a^	13.50±0.1	0.13±0.01	5.63±0.2	42.65
PZA^a^	0.70±0.03	0.82±0.02	0.43±0.03	0.52
**C133A/F68W**				
NAM^a^	22.90±0.2	0.19±0.01	9.82±0.02	52.8
PZA^a^	7.43±0.1	0.83±0.03	5.21±0.01	6.28

a Reactions were carried out following the standard spectrophotometric method.

b Reactions were analyzed by standard HPLC method.

In order to study critical amino acids that participate in substrate specificity and activity, seven mutants were generated by site-directed mutagenesis (Table 1): two for residues involved in the catalysis (K104 and Q96), one for the fourth residue involved in metal binding (E65), one for the residue forming hydrogen bonds between the main and lateral chains (T12), one for the residue involved in the *cis*-peptide bond and in the formation of the oxyanion hole (C133), and one for the residue forming one of the faces of the active site (F68). Specific activity and kinetic parameters of the 7 mutants were determined for NAM and NAM analogues (Table 1). Mutants T12Q and K104A were expressed as insoluble inclusion bodies. Although these two mutants were solubilized, they showed a marked reduction in specific activity towards their natural substrate NAM compared with wild type (1.58% and 1.45%, respectively), probably due to non-proper refolding. Two changes were generated at position 96 (Q96K and Q96A), since this residue lies close to the catalytic lysine of the crystallized nicotinamidases [9], [35], [36], [37], [54], [57]. However, this mutation in OiNIC reduced, but did not abolish, the activity towards NAM. Q96K showed 37.3% specific activity, while Q96A maintained almost full activity, probably because alanine modifies the conformation of the active site to a lesser extent. Both mutants (Q96K and Q96A) also exhibited a lower *K_m_* for methylnicotinate than the wild-type. Mutant Q96A also showed a remarkably higher *k_cat_* and *k*_cat_/*K_m_* for 5-methylnicotinamide (15.9±0.2 s^−1^ and 29.04 mM^−1^s^−1^, respectively) and for methylnicotinate (0.6 s^−1^±0.05and 1.17 mM^−1^s^−1^, respectively) (Table 1). This residue somehow modifies the active center, broadening the specificity of the enzyme, but it is not an essential residue for catalysis.

Surprisingly, mutant C133A showed a lower *K_m_* for PZA (0.36±0.02 mM *vs* 0.81±0.01 mM of the wild-type) and ethylnicotinate (0.34±0.01 mM *vs* 1.02±0.01 mM). It also showed greater *k_cat_* and *k_cat_*/*K_m_* value than the wild-type for PZA, 5-methylnicotinamide, methylnicotinate and ethylnicotinate (Table 1). Mutation F68W also affected the active site cavity, and enhanced the binding of pyrazinamide, increasing the affinity of OiNIC for PZA (*K_m_* 0.5±0.02 mM) compared to the wild-type, improving the *k_cat_/K_m_* 2.5-fold (7.9 mM^−1^s^−1^). This mutation also reduced the *K_m_* for the natural substrate NAM 2-fold to 0.17±0.01 mM. Based on the above, a doubled mutant (C133A/F68W) combining the two latter mutations was constructed in order to obtain more activity towards PZA. However, no improvement in the catalytic efficiency compared with single mutants was observed, since both changes gave rise to a higher *k*_cat_ for PZA (5.21±0.01 s^−1^) but also a higher *K_m_* (0.83±0.03 mM).

Finally, mutation at position E65H, which affects the fourth residue involved in ion metal binding, produced a dramatic loss in the catalytic efficiency for PZA (0.52 mM^−1^s^−1^), without changing its *K_m_* (0.82±0.02 mM). Surprisingly, this mutant showed the lowest *K_m_* for NAM among the enzymes shown in Table 1.

### Metal Binding

Nicotinamidases accommodate a metal ion in the active center cavity, which actively participates in the orientation of the substrate and in the conversion of NAM to NA. However, different nicotinamidases present different metal ions. While *P. horikoshii, S. pneumoniae* and *A. baumanii* nicotinamidases bind a Zn^2+^ ion [1], [35], [36], *M. tuberculosis* nicotinamidase contains Mn^2+^/Fe^2+^ or Fe^2+^ in its structure [3], [37]. Inductively Coupled Plasma-Optical Emission Spectrometry (ICP-OES) confirmed the presence of Zn^2+^ in the active center of OiNIC in a molecular ratio of 1∶1 (protein:metal ion). The enzyme contained 235.2±0.05 µM of Zn^2+^ in 241.7 µM protein, while it only contained 3.21±0.01 µM of Fe^2+^ and 0.76±0.01 µM Mn^2+^.

### Inhibition by Nicotinaldehydes

Nicotinaldehydes have been reported to act as good competitive inhibitors for several nicotinamidases [1]. Thus, OiNIC was tested with nicotinaldehyde and 5-bromo-nicotinaldehyde. Doubled-reciprocal plots confirmed the competitive nature of the inhibition with nicotinamide in both cases (Fig. S3A and B). The *K_i_* values for the inhibition of nicotinaminidase were calculated using the Morrisońs quadratic equation since, in both cases, they were lower than 5 µM (3.4 µM for nicotinaldehyde and 4.4 µM for 5-Br-nicotinaldehyde; Fig. S3C). These *K*_i_ values were similar to those found in *S. cerevisiae* Pnc1 (1.4 µM and 4 µM, respectively), but higher than those corresponding to the enzymes from *P. falciparum, S. pneumoniae* and *Clostridium burgdorferi,* with *K*_i_ values in the nM range [1].

### Structural Analysis

**Figure 4:**
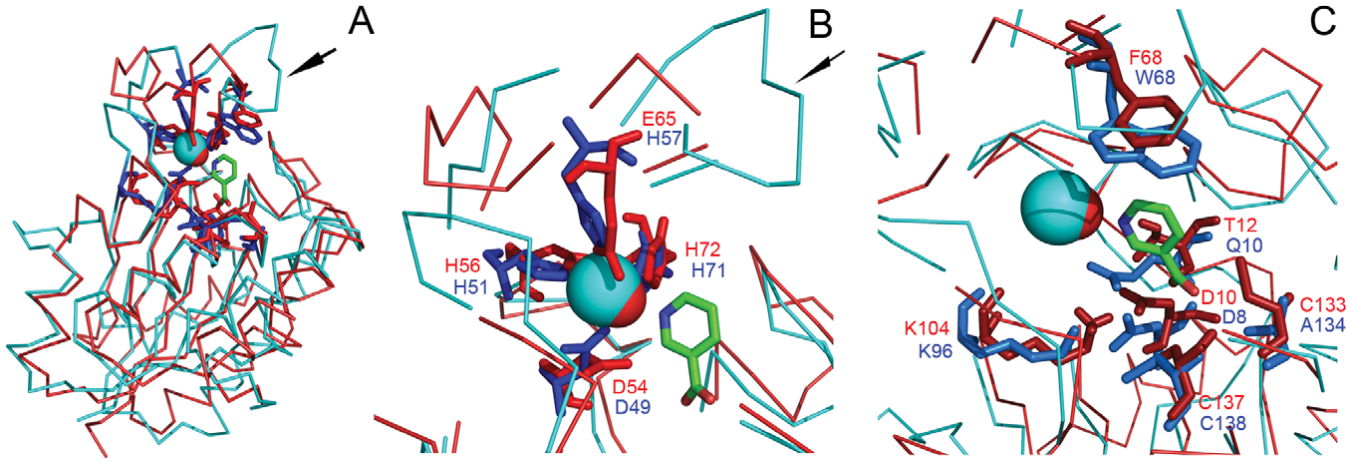
Structural alignment of modelled OiNIC and crystallized *Mycobacterium tuberculosis* MtPncA. A) Monomer of OiNIC is represented in red and monomer of MtPncA in blue. Fe^2+^ and Zn^2+^ are represented as blue and red spheres, respectively. The arrow represents the 51–71 loop of MtPncA. NAM is colored in green. B) Residues interacting with the metal ion. C) Residues forming the active site cavity and interacting with NAM.

**Figure 5:**
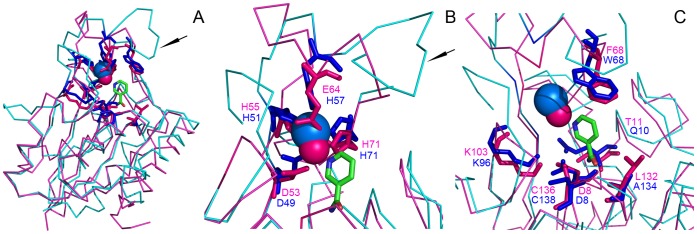
Structural alignment of crystallized SpNIC and crystallized *M. tuberculosis* MtPncA. A) Monomer of SpNIC is represented in pink and monomer of MtPncA in blue. Fe^2+^ and Zn^2+^ are represented as blue and pink spheres, respectively. NAM is colored in green. B) Residues interacting with the metal ion. C) Residues forming the active site cavity and interacting with NAM.

The crystallized structure of *S. pneumoniae* nicotinamidase (PDB code: 3O90, 53% identity) [54] was selected as a template by Geno 3D [49] to create an OiNIC model (Fig. 4A and S4). The enzyme folds as a typical α/β protein with five-stranded β-sheets flanked by three helices on one side (α5, α6 and α8) and four helices on the opposite side (α1, α2, α4 and α7), as it also occurred in SpNIC structure (Fig. 5A, pink backbone). This structure is characteristic of the isochorismatase-like hydrolases superfamily, which includes nicotinamidases/pyrazinamidases, *N*-carbamoylsarcosin amido hydrolases, YecD proteins, YcaC proteins, and PhzD proteins. These enzymes share a common structural fold, but catalyse different reactions in separate biochemical pathways. The active site of the modelled OiNIC was located in a solvent-accessible pocket formed primarily by three loop regions containing residues 10–22 (between β1 and α1), residues 104–112 (between β3 and α5), and residues 133–137 (between β4 and α6) (OiNIC numbering) (Fig. S4).

### Phylogenetic Analysis

**Figure 6:**
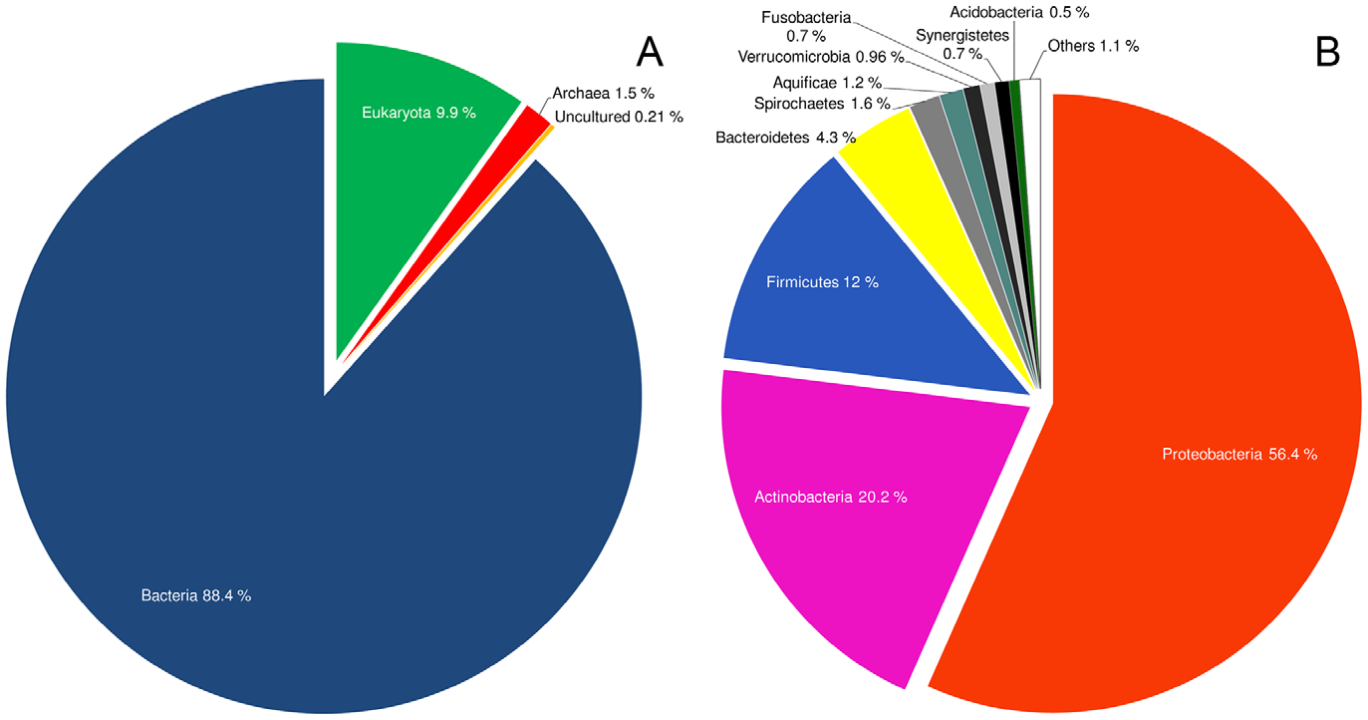
Nicotinamidase distribution (A) in biology and (B) among bacteria.

To obtain further information on the origin of OiNIC, sequence analysis was carried out, since to the best of our knowledge, such a detailed phylogenetic analysis of nicotinamidases has not been realized before. Nicotinamidases are widely distributed across biology, but most have been found in bacterial genomes, representing 88% of all known nicotinamidase sequences (Fig. 6A). The rest include some Eukaryotes (10%), mainly belonging to the phylum Fungi (6.4%), and a minority to Archaea (1.5%) (Fig. 6A). Among bacteria (Fig. 6B), more than half of the sequences found (56.4%) belong to Proteobacteria, such as the crystallized one from *Acinetobacter baumanii* [36]. Nicotinamidases from phylum Firmicutes, where OiNIC and *Streptococcus pneumoniae* [54] nicotinamidase are included, represent about 12% of bacterial enzymes, whereas *Mycobacterium* and other actinobacteria nicotinamidases represent about 20%. Examples of nicotinamidases can be found in almost every other bacterial phylum, the most abundant being the examples in phylum Bacteroidetes/Chlorobi, Spirochaetes and Aquificae with 4.3%, 1.6% and 1.2%, respectively.

**Figure 7:**
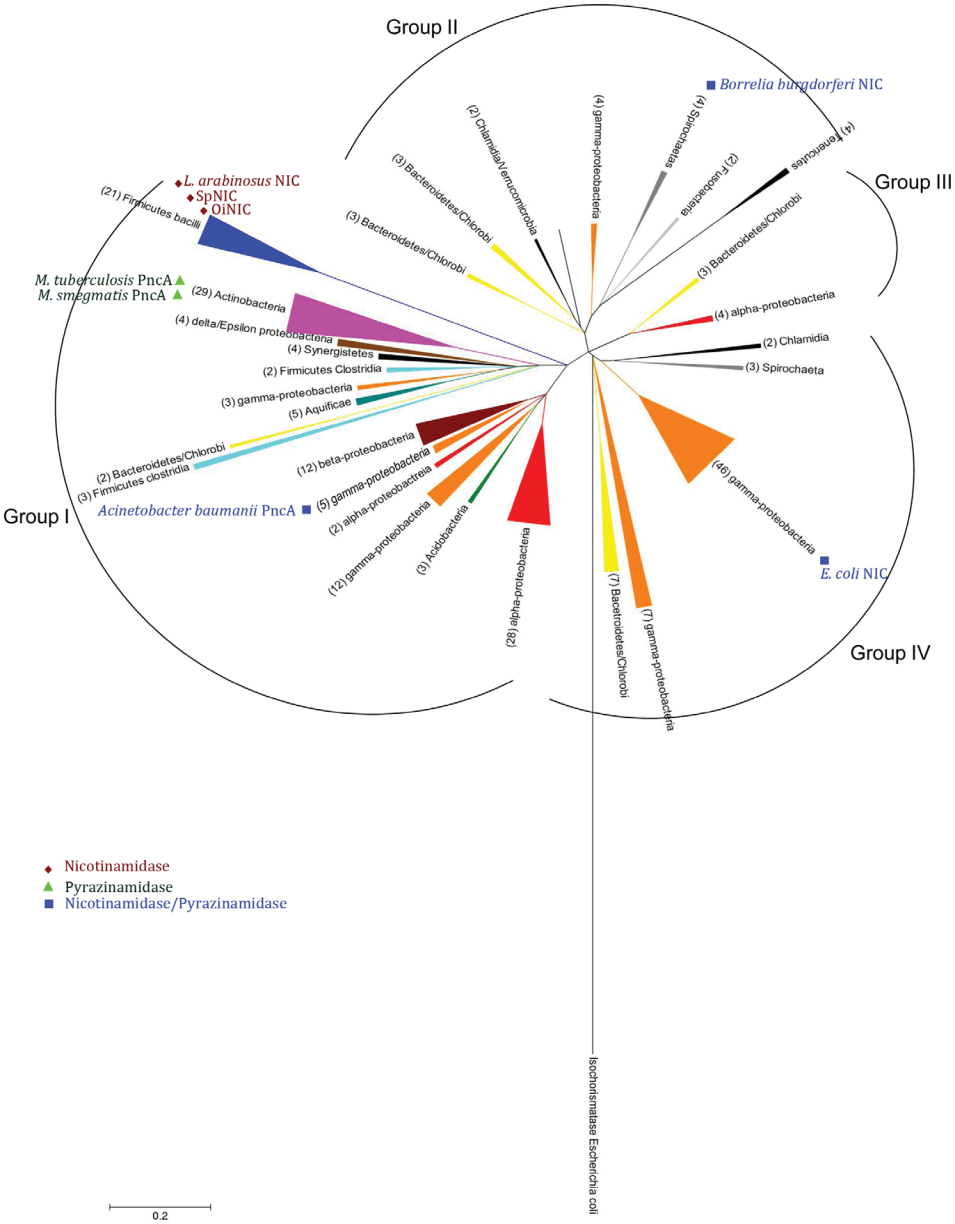
Phylogenetic distribution of bacterial nicotinamidases. For reasons of clarity, branches are shown compressed as triangles. The scale bar at the lower left indicates the rate of amino acids substitutions. The triangle base corresponds to the number of compressed sequences involved, which is also shown in parentheses. The triangle height corresponds to evolutionary distance. The bacterial nicotinamidase sequences used in this study (see text for details) are phylogenetically divided into 4 groups, in which biochemically characterized nicotinamidases are positioned according with its activity towards nicotinamide and/or pyrazinamide. *E. coli* isochorismatase was used as outgroup. The phylogenetic tree was obtained using MEGA 5.0 [53].

A phylogenetic tree was constructed with MEGA 5.0 [53], restricting the sequences retrieved from the Uniprot database to the gene ontology term “nicotinamidase” or “pyrazinamidase”, and just one strain from each species (Fig. 7). The tree revealed that the phylogenetic relationships in the nicotinamidases are quite different from the traditional bacterial phylogeny described in the Tree of Life (http://tolweb.org), and showed four groups in the phylogenetic tree (Fig. 7). Group I included all Gram+ bacteria from phylum Firmicutes and Actinobacteria, where OiNIC and the rest of nicotinamidases from class Bacilli clustered together. However, Firmicutes from class Clostridia appeared separated into two branches. In addition, group I also contained a few branches formed by Gram– bacteria from phyla Spirochaetes, Bacteroidetes/Chlorobi, Aquificae, Actinobacteria, Synergistetes and many examples of Proteobacteria, clustering together with the above mentioned Gram+ species. The distribution of Proteobacteria in the tree was very divergent, and examples of Proteobacteria were found in all groups. The same phenomenon occurred with phylum Bacteroidetes and Spirochaetes, examples of these phyla appearing in groups II, III and IV.

It seems that only in the case of Firmicutes class Bacilli and Actinobacteria has the evolution of nicotinamidases been vertical, and it is conserved at phylum level, whereas in the case of Proteobacteria, different origins for this enzyme are evident. This could explain the wide distribution of Proteobacteria nicotinamidases (56% of bacterial nicotinamidases, Fig. 6B). Since *Oceanobacillus iheyensis* belongs to the phylum Firmicutes, an extensive study of the sequences of Firmicutes nicotinamidases was carried out, and compared with that of 16S rRNA sequences (Fig. S5 and S6). As expected, the results pointed to an overall evolution of these nicotinamidases in parallel with the evolution of bacterial species. However, some evidence of horizontal gene transfer events appeared. The cluster comprising the nicotinamidases of the genus *Lactobacillus* clustered together with the nicotinamidases of the class Bacillales (*Bacillus, Oceanobacillus, Geobacillus*) rather than with the nicotinamidases from *Streptococcus*.

## Discussion

Nicotinamidases have proved to be relevant enzymes in pharma and biotechnology. This paper describes the cloning, overexpression and a detailed characterization of a new nicotinamidase gene from the extremophilic microorganism *Oceanobacillus iheyensis* HTE831. The recombinant enzyme expressed into *E. coli* Rosetta 2 (OiNIC) showed an optimum pH of around pH 6.0–6.5, and was found to be stable from acid to neutral pHs. OiNIC also showed good thermostability, with a *T_m_* value of 53.3±0.2°C at pH 7.3, making it about 10°C more thermostable than the *Mycobacterium tuberculosis* nicotinamidase (MtPncA), whose *T_m_* was 43°C [3]. OiNIC *T_m_* was further improved by the addition of a protein stabilizer, such as ammonium sulfate, which increased the *T_m_* up to 61.34±0.2°C, or the addition of a competitive inhibitor, nicotinaldehyde, which increased the *T_m_* by 6°C (*T_m_:* 59.7±0.3°C), suggesting strong binding of the inhibitor to OiNIC. These results indicate that this technique could be very useful for the high-throughput discovery of novel therapeutic inhibitors or analogues of nicotinamidases using chemical libraries.

OiNIC, which binds Zn^2+^ in the active center, was a good NAM catalyst (*k_cat_* 11.6±0.1 s^−1^) and was active towards different nicotinamide analogues, including the pro-drug pyrazinamide, used in tuberculosis treatment. Substitutions at the 5-position were well-tolerated, showing 38% of NAM specific activity, almost 3-fold more than for pyrazinamide. However, nicotinate esters were not so good as substrates, representing only about 0.5% (ethylnicotinate) and 1.8% (methylnicotinate) of the activity towards NAM. This substrate specificity was similar to that of other nicotinamidases [1]. The *K_m_* values for NAM and PZA were in the range described in the bibliography, which varies from 0.0002 mM to 0.65 mM for NAM [1], [5], [58] and from 0.056 to 0.63 mM for pyrazinamide [2], [5]. However, the *K_m_* values obtained in OiNIC for NAM were closer to those previously described for non-pathogenic nicotinamidases, whereas the *K_m_* for PZA was slightly higher than that described in the bibliography (0.1–0.4 mM), suggesting that this enzyme is more like a nicotinamidase than a pyrazinamidase. However, its *k*_cat_ for PZA (2.6±0.1 s^−1^) was more than 50 times greater than the *k*_cat_ for PZA of the nicotinamidase/pyrazinamidase from *Acinetobacter baumanii* [36].

The catalytic efficiency of OiNIC towards NAM was 43.48 mM^−1^ s^−1^ (Table 1), which is 2-fold higher compared with *Caenorhabditis elegans* CePNC1 [1], but lower than described for *S. pneumoniae* NIC (SpNIC), *M. tuberculosis* PncA (MtPncA) and *S. cerevisiae* PncA [1]. On the PZA side, the catalytic efficiency of OiNIC (3.20 mM^−1^ s^−1^) (Table 1) was 6-fold higher than that of *A. baumanii* [36], but 3-fold lower than that of *M. tuberculosis* [1]. These data indicated that the catalytic efficiencies of OiNIC were in the normal range for nicotinamidases, especially those from non-pathogenic microorganisms.

Mutants of selected residues were designed to find critical amino acids for catalysis and metal binding (see Table 1 and Fig. 4). The results showed that K104 was a part of the catalytic triad; that E65 was a crucial metal binding residue, and that C133 and F68 were residues involved in the substrate specificity of OiNIC since they modify the shape and volume of active center. In fact, mutation C133A changed the specificity of OiNIC, suggesting that this residue could be involved in the binding of nicotinamide or pyrazinamide in the active site, since this mutation increased the catalytic efficiency of OiNIC towards PZA 2.9-fold compared with the wild-type OiNIC (Table 1). Mutation F68W also affected the active site cavity and improved the binding of pyrazinamide, increasing not only the affinity of OiNIC for PZA (*K_m_* 0.5±0.02 mM) compared with the wild-type enzyme, but also the *k_cat_/K_m_* (7.9 mM^−1^s^−1^) 2.5-fold.

These results were more evident when modelled OiNIC was structurally aligned with crystallized MtPncA (Fig. 4) [37]. The first structural difference observed was the absence of the protrusion described for MtPncA (Fig. 4A, arrow), corresponding to its 51–71 loop, which occludes the mouth of the binding cavity [37]. This loop region appears to be specific for the correct positioning of the fourth residue involved in the coordination of metal ion (Zn^2+^ or Fe^2+^), which could be glutamic (E), serine (S) or histidine (H) (Table S2). This papers shows that, apart from the two motifs described of this region by Petrella et al. [37], **D**W**H**PXX**H** (where X represents a non-conserved residue) for PhPncA and AbPncA, and **D**F**H**XXPXX**H** for *M. tuberculosis* PncA, another **D**A**H**XXXDXXHP**E** motif (D54–E65 region in OiNIC) exists in Firmicutes nicotinamidases, such as SpNIC and OiNIC (Fig. 1, Fig. 4 and 5, arrow). This sequence makes it possible, not only in the modelled OiNIC but also and more clearly in the crystallized SpNIC (PDB code:3094) to position the fourth residue (glutamic acid, E64) correctly at 2.1 Å from Zn^2+^ and also 3.2 Å from the N1 of nicotinic acid found in the SpNIC crystal [54] (Fig. 5B). These distances agree with the 2.3 Å found between H57 (NE2) and Fe ^2+^ in crystallized MtPncA [37] (PDB code: 3Pl1). To achieve this distance in MtPncA, the 51–57 loop pulls the histidine to accommodate the bigger Fe^2+^ ion (Fig. 5C). The second structural difference lies in the lid amino acid of the cavity, the tryptophan W68 in MtPncA and phenylalanine F68 in Firmicutes (Fig. 4C and 5C), which is placed at the top site of the cavity. This amino acid was seen to be critical for substrate specificity, improving the binding of pyrazinamide, as occurs in F68W OiNIC mutant. This tryptophan is also part of AbPncA active site (W86) [36], but differs from those of MtPncA and Firmicute nicotinamidases in the presence of a ‘gate region’ between β3 and β4, which permits a strong substrate binding in AbPncA compared to other nicotinamidases [59].

The two structural differences shown in this paper suggest biochemically different phylogenetic origins for nicotinamidases and pyrazinamidases. When the characterized nicotinamidases and pyrazinamidases were inserted in Figure 7, no clear conclusion was evident. However, when the sequence region corresponding to the four amino acids involved in the ion binding was aligned (Fig. S2), two distinct phylogenetic origins were found. One corresponded to the above mentioned **D**W**H**PXX**H** for AbPncA and PhPncA, which also included *Borrelia burgdorferi* NIC (BbNIC) and *Escherichia coli* NIC (EcNIC). The second included motifs found in MtPncA and Firmicutes (OiNIC and SpNIC) with a clear distinction between that of Firmicutes with an E and that of *Mycobacterium* with an H as the fourth amino acid involved in metal binding. In addition, this partial alignment (Fig S7) shows that the sequence of this metal binding region is flanked in all cases by **D**X**H** at the N-terminal and by PX**H** at the C-terminal. These conserved sequences pointed to a clear nicotinamidase/pyrazinamidase fingerprint, which together with the **C**V sequence, where C is the catalytic cysteine (Fig. 1), could be used to assign new sequences as nicotinamidases/pyrazinamidases.

In conclusion, the classification of nicotinamidases carried out in this paper reveals for the first time their distribution in biology and the phylogenetic relationships between bacterial nicotinamidases. The fact that proteins from phylogenetically distant species cluster together points to horizontal gene transfer events. Much work must be done to fully understand the divergence among bacterial nicotinamidases and the characteristics of each group of species, data that would broaden our understanding as to which structural characteristics explain the greater activity towards NAM than PZA, or the presence of one or other metal ion in the active center.

## Supporting Information

Figure S1 **Nicotinamidase reaction.** Nicotinamidases hydrolyzes nicotinamide to give nicotinic acid and ammonia. The last compound was coupled with glutamate dehydrogenase to follow spectrophotometrically nicotinamidase activity by monitoring the decrease in absorbance of NAD(P)H^+^. This coupled enzyme assay has been used previously to follow sirtuin activity [34], which renders deacetylated peptide/protein, O-acetyl-ADPribose and nicotinamide.
(TIF)

Figure S2 **SDS-PAGE of the pure OiNIC and enzyme cross-linked with dimethylsuberimidate.** M: molecular weight standards (New England Biolabs: P7708S). Lane 1: Purified OiNIC. Lane 2: Purified OiNIC with DMS (3 mg/mL). Protein monomer is about 21 kDa, protein dimer is about 42 kDa.
(TIF)

Figure S3 **Inhibition of OiNIC by nicotinaldehydes.** The Lineweaver-Burke plots for competitive inhibition by nicotinaldehyde (A) and 5-Bromo-nicotinaldehyde (B). Inhibition reactions (1 mL) contained 0.3 mM NADPH, 10 mM α-ketoglutarate, 9.7 µg GDH, 1.3 µg of OiNIC in 100 mM sodium phosphate pH 7.3, and increasing concentrations of NAM in the presence of 0 µM (filled symbol), 10 µM (open symbol) and 20 µM (grey symbol) of corresponding inhibitor at 37°C. C) Relative inhibition of OiNIC by nicotinaldehyde (•) and 5-Bromo-nicotinaldehyde (▪). The reactions at 37°C were carried out in the presence of 1 mM NAM and different concentrations of the inhibitor in the same conditions as above. Morrison’s equation was used to fit data and to obtain the *K_i_* value, as described in Materials and Methods.
(TIF)

Figure S4 **Modelled structure of OiNIC.** Zn^2+^ atom is shown as a sphere and nicotinic acid as sticks.
(TIF)

Figure S5 **Phylogenetic distribution of nicotinamidases from phylum Firmicutes.** The phylogenetic tree was obtained using MEGA 5.0 [53].
(TIF)

Figure S6 **Phylogenetic distribution 16S rRNA of the Firmicutes microorganisms.** The species were the same as those used in Fig. S5. The phylogenetic tree was obtained using MEGA 5.0 [53]
(TIF)

Figure S7 **Phylogenetic distribution of characterized nicotinamidases/pyrazinamidases based on partial sequence alignment of conserved motifs.** OiNIC: *Oceanobacillus iheyensis* nicotinamidase; LaNIC: *Lactobacillus arabinosus* nicotinamidase; SpNIC_3o90: *Streptococcus pneumoniae* nicotinamidase; MtPncA_3pL1: *Mycobacterium tuberculosis* pyrazinamidase; MsPncA: *Mycobacterium smegmatis* pyrazinamidase; AbPncA_2wt9: *Acinetobacter baumanii* nicotinamidase/pyrazinamidase; *Borrelia burgdorferi* nicotinamidase/pyrazinamidase; PhPncA_1ilw: *Pyrococcus horikoshii* nicotinamidase/pyrazinamidase; EcNIC: *Escherichia coli* nicotinamidase/pyrazinamidase. Stars represent the four amino acids involved in metal binding. Top sequence and bars represent consensus conservation of each residue. This figure was obtained using Chimera program [60].
(TIF)

Table S1 **Oligonucleotide sequences used for site-directed mutagenesis.**
(PDF)

Table S2 **Comparative study of the residues involved in nicotinamidase activity.**
(PDF)
