# Supplementary figures and images for: Biochemical and Mutational Analysis of a Novel Nicotinamidase from Oceanobacillus iheyensis HTE831
Source: PLoS One. 2013 Feb 25;8(2):e56727. doi: 10.1371/journal.pone.0056727 (PMC3581539; doi:10.1371/journal.pone.0056727)

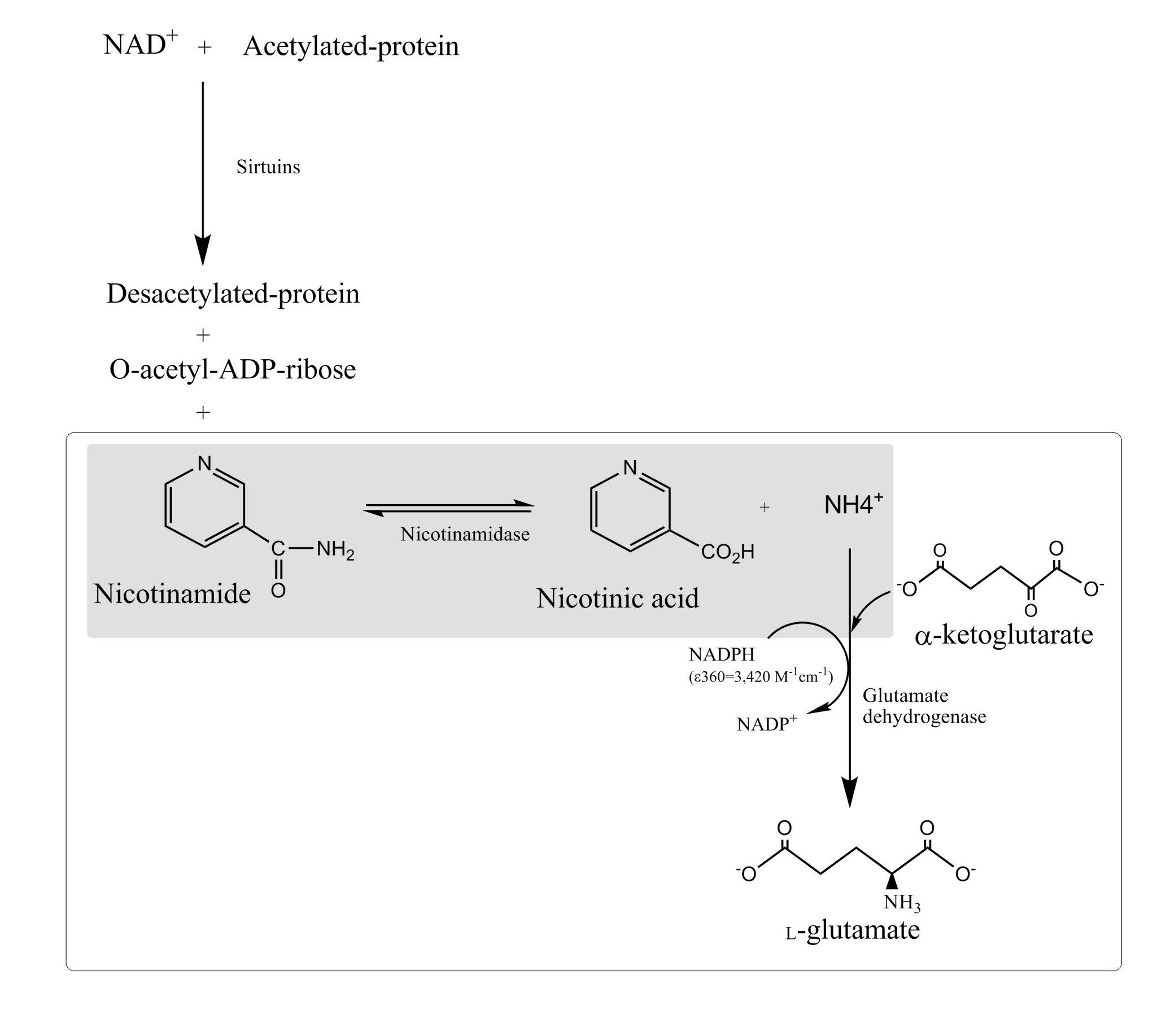

Supplement: Figure S1 — Nicotinamidase reaction. Nicotinamidases hydrolyzes nicotinamide to give nicotinic acid and ammonia. The last compound was coupled with glutamate dehydrogenase to follow spectrophotometrically nicotinamidase activity by monitoring the decrease in absorbance of NAD(P)H+. This coupled enzyme assay has been used previously to follow sirtuin activity [34], which renders deacetylated peptide/protein, O-acetyl-ADPribose and nicotinamide. (TIF) [file pone.0056727.s001.tif]

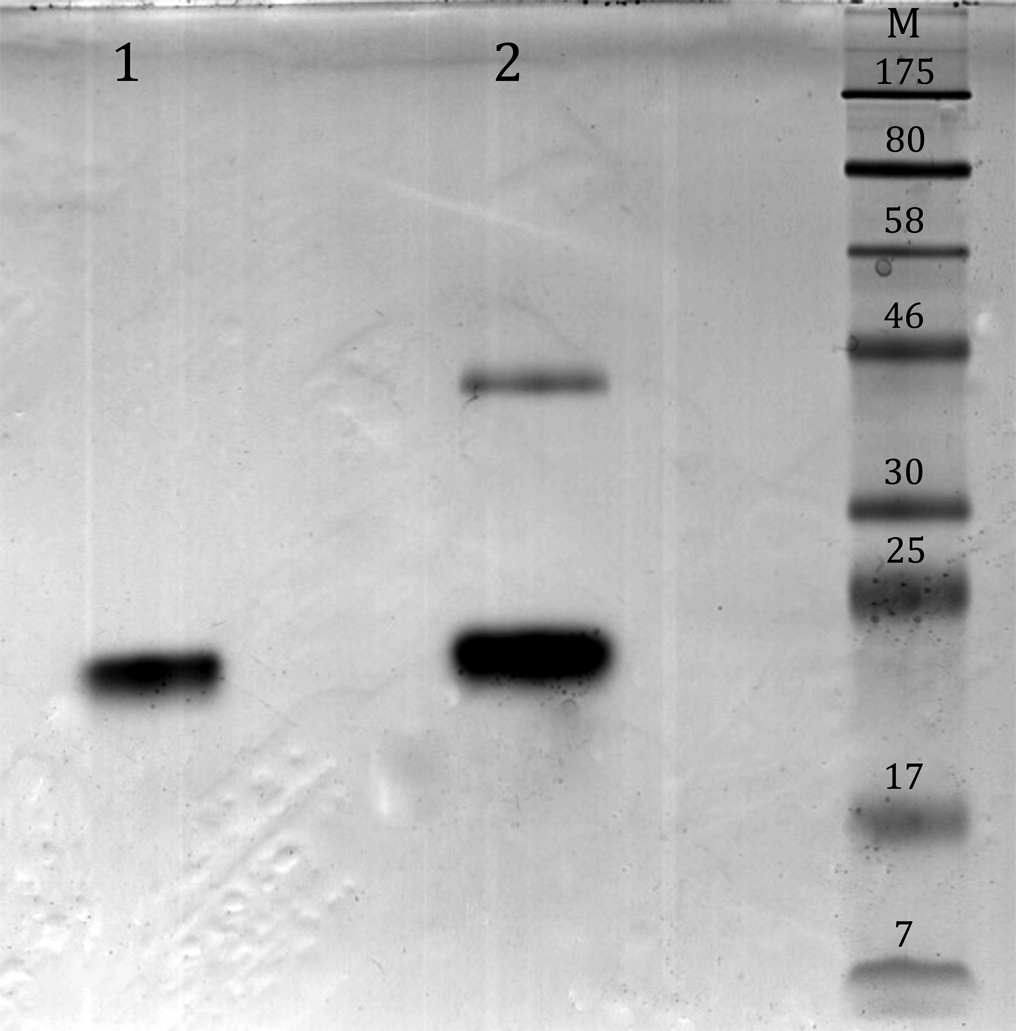

Supplement: Figure S2 — SDS-PAGE of the pure OiNIC and enzyme cross-linked with dimethylsuberimidate. M: molecular weight standards (New England Biolabs: P7708S). Lane 1: Purified OiNIC. Lane 2: Purified OiNIC with DMS (3 mg/mL). Protein monomer is about 21 kDa, protein dimer is about 42 kDa. (TIF) [file pone.0056727.s002.tif]

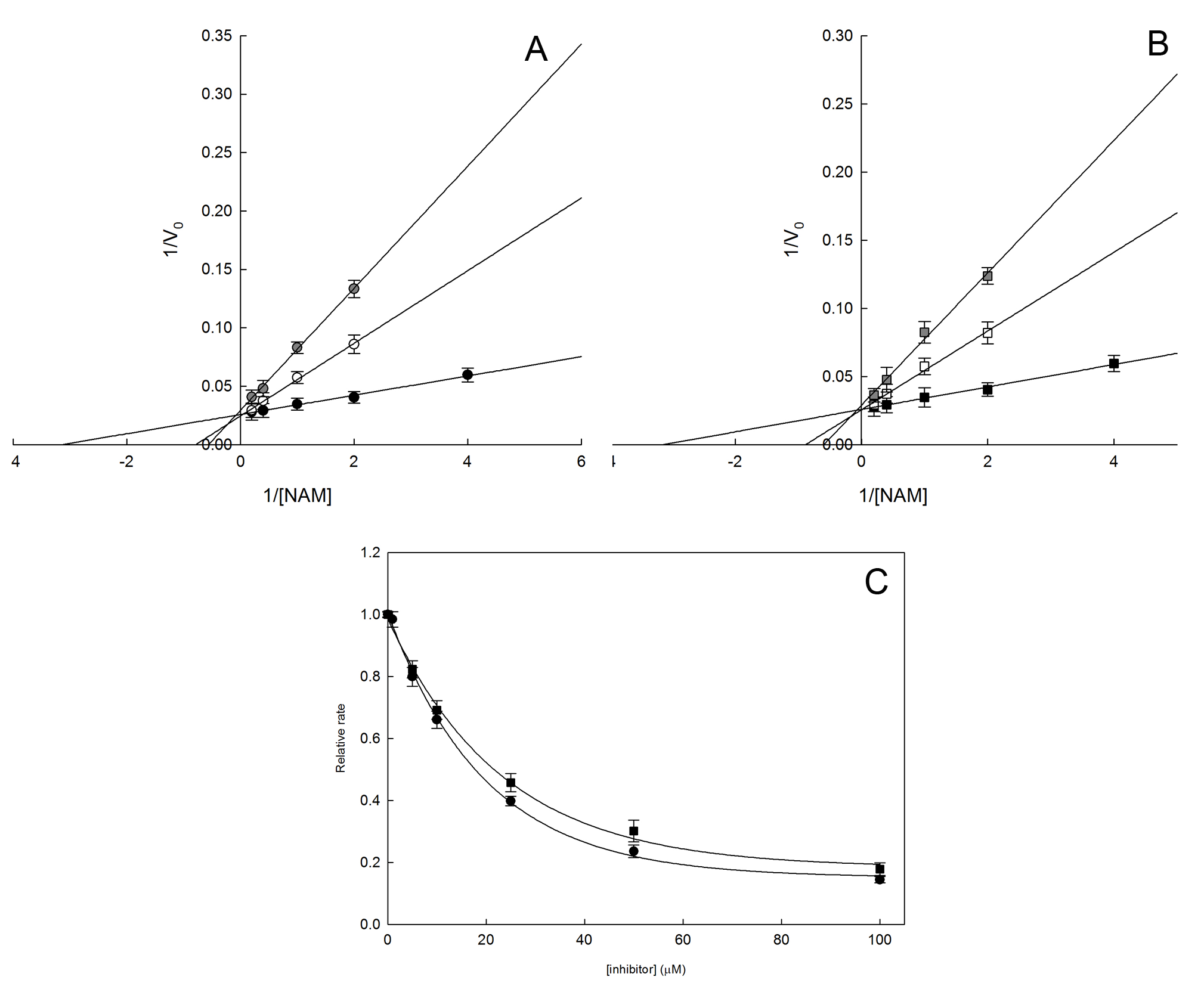

Supplement: Figure S3 — Inhibition of OiNIC by nicotinaldehydes. The Lineweaver-Burke plots for competitive inhibition by nicotinaldehyde (A) and 5-Bromo-nicotinaldehyde (B). Inhibition reactions (1 mL) contained 0.3 mM NADPH, 10 mM α-ketoglutarate, 9.7 µg GDH, 1.3 µg of OiNIC in 100 mM sodium phosphate pH 7.3, and increasing concentrations of NAM in the presence of 0 µM (filled symbol), 10 µM (open symbol) and 20 µM (grey symbol) of corresponding inhibitor at 37°C. C) Relative inhibition of OiNIC by nicotinaldehyde (•) and 5-Bromo-nicotinaldehyde (▪). The reactions at 37°C were carried out in the presence of 1 mM NAM and different concentrations of the inhibitor in the same conditions as above. Morrison’s equation was used to fit data and to obtain the Ki value, as described in Materials and Methods. (TIF) [file pone.0056727.s003.tif]

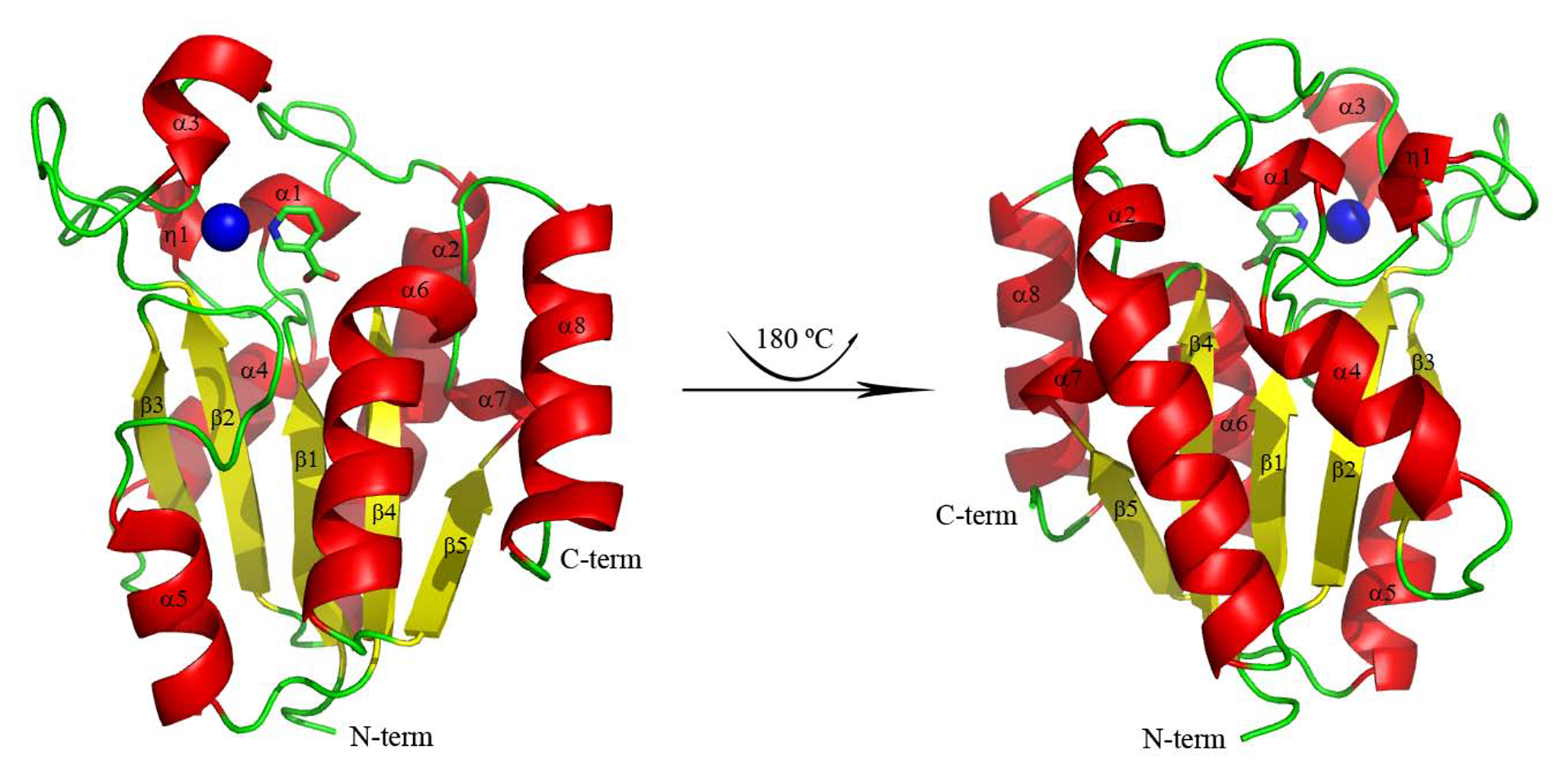

Supplement: Figure S4 — Modelled structure of OiNIC. Zn2+ atom is shown as a sphere and nicotinic acid as sticks. (TIF) [file pone.0056727.s004.tif]

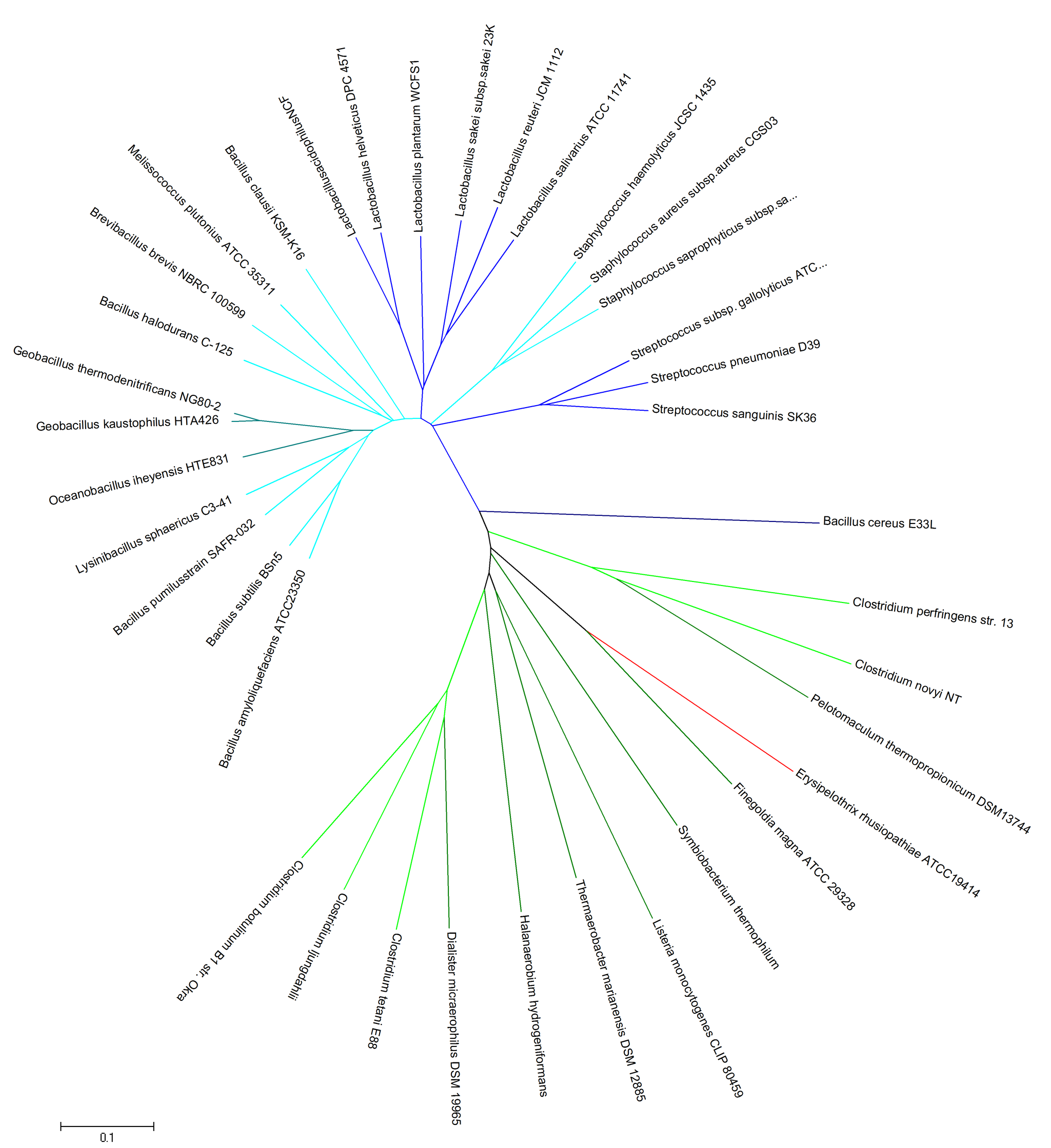

Supplement: Figure S5 — Phylogenetic distribution of nicotinamidases from phylum Firmicutes. The phylogenetic tree was obtained using MEGA 5.0 [53]. (TIF) [file pone.0056727.s005.tif]

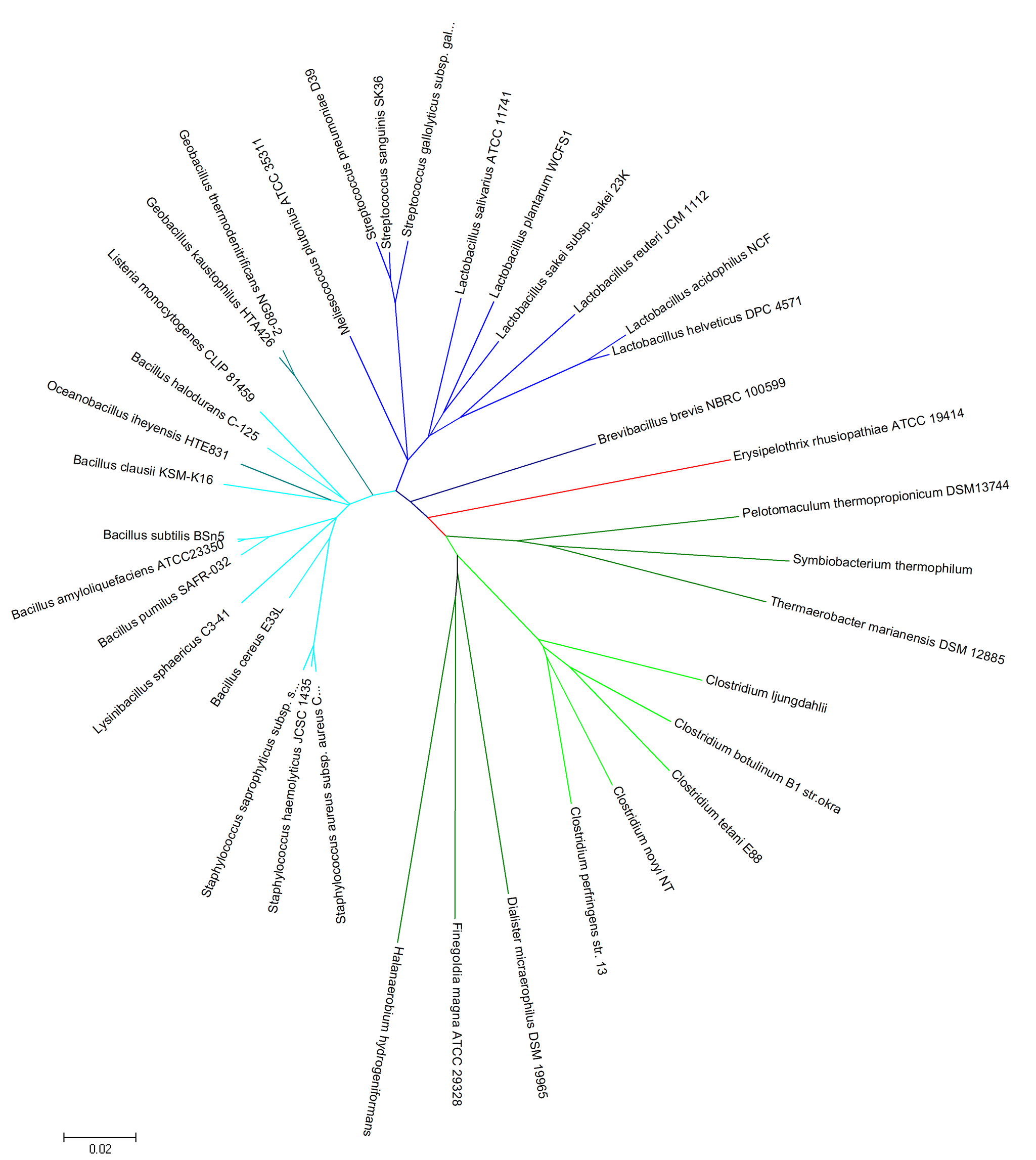

Supplement: Figure S6 — Phylogenetic distribution 16S rRNA of the Firmicutes microorganisms. The species were the same as those used in Fig. S5. The phylogenetic tree was obtained using MEGA 5.0 [53] (TIF) [file pone.0056727.s006.tif]

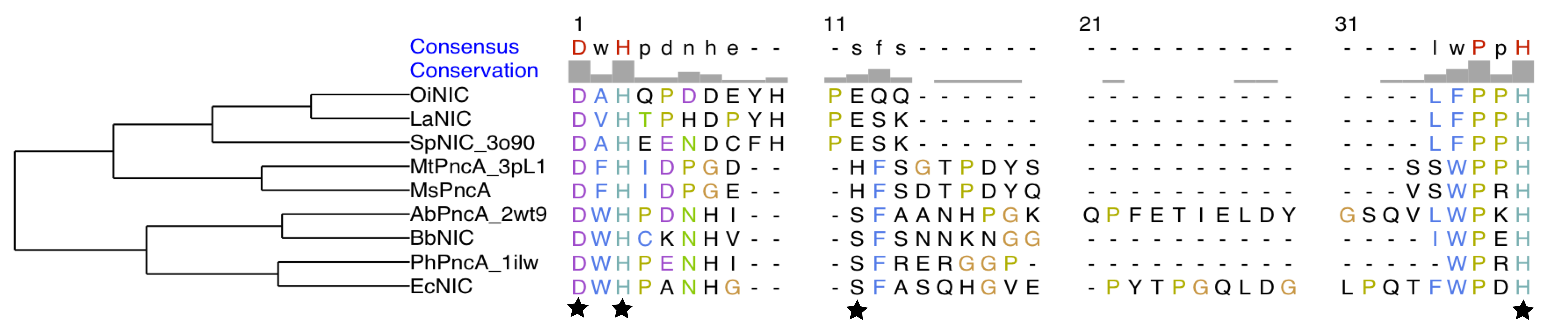

Supplement: Figure S7 — Phylogenetic distribution of characterized nicotinamidases/pyrazinamidases based on partial sequence alignment of conserved motifs. OiNIC: Oceanobacillus iheyensis nicotinamidase; LaNIC: Lactobacillus arabinosus nicotinamidase; SpNIC_3o90: Streptococcus pneumoniae nicotinamidase; MtPncA_3pL1: Mycobacterium tuberculosis pyrazinamidase; MsPncA: Mycobacterium smegmatis pyrazinamidase; AbPncA_2wt9: Acinetobacter baumanii nicotinamidase/pyrazinamidase; Borrelia burgdorferi nicotinamidase/pyrazinamidase; PhPncA_1ilw: Pyrococcus horikoshii nicotinamidase/pyrazinamidase; EcNIC: Escherichia coli nicotinamidase/pyrazinamidase. Stars represent the four amino acids involved in metal binding. Top sequence and bars represent consensus conservation of each residue. This figure was obtained using Chimera program [60]. (TIF) [file pone.0056727.s007.tif]
