# Supplementary material for: Biochemical and Mutational Analysis of a Novel Nicotinamidase from Oceanobacillus iheyensis HTE831
Source: PLoS One. 2013 Feb 25;8(2):e56727. doi: 10.1371/journal.pone.0056727 (PMC3581539; doi:10.1371/journal.pone.0056727)
Supplement: Table S1 — Oligonucleotide sequences used for site-directed mutagenesis. (PDF) [file pone.0056727.s008.pdf]

Table S1. Oligonucleotide sequences used for site-directed mutagenesis

OiNIC Mutant	Primers
T12Q	5’ GGCATTATTAAATATCGATTATCAAATTGATTTTGTAGCAGAAGATGG
	3’ CCATCTTCTGCTACAAAATCAATTTGATAATCGATATTTAATAATGCC
Q96K	5’CACTTTATCAACGTATAAAAGAAAAGGAAAATGTCTATTACTTTGATAA
	3’ TTATCAAAGTAATAGACATTTTCCTTTTCTTTTATACGTTGATAAAGTG
Q96A	5’ CACTTTATCAACGTATAAAAGAAGCGGAAAATGTCTATTACTTTGATAA
	3’ TTATCAAAGTAATAGACATTTTCCGC TTCTTTTATACGTTGATAAAGTG
K104A	5’ AGGAAAATGTCTATTACTTTGATGCAACAAGATATAGTGCATTTGCTGG
	3’ CCAGCAAATGCACTATATTTGTTGCATCAAAGTAATAGACATTTTCCTG
C133A	5’ GAAGTTCATCTTGTTGGAGTTGCTACTGATATATGTGTTTTACATAC
	3’ GTATGTAAAACACATATATCAGTAGCAACTCCAACAAGATGAACTTC
F68W	5’ GTATCATCCCGAACAACAACTATGGCCTCCCCATAATATAGTTGGAAC
	3’ GTTCCAACTATATTATGGGGAGGCCATAGTTGTTGTTCGGGATGATAC
E65H	5’ AACCTGACGATGAGTATCATCCCCACCAACAACTATTTCCTCCCCATAA
	3’TATGGGGAGGAAATAGTTGTTGGTGGGGATGATACTCATCGTCAGGTTG
C133A-F68W (template C133A)	5’ GTATCATCCCGAACAACAACTATGGCCTCCCCATAATATAGTTGGAAC
	3’ GTTCCAACTATATTATGGGGAGGCCATAGTTGTTGTTCGGGATGATAC
