# Supplementary material for: Biochemical and Mutational Analysis of a Novel Nicotinamidase from Oceanobacillus iheyensis HTE831
Source: PLoS One. 2013 Feb 25;8(2):e56727. doi: 10.1371/journal.pone.0056727 (PMC3581539; doi:10.1371/journal.pone.0056727)
Supplement: Table S2 — Comparative study of the residues involved in nicotinamidase activity. (PDF) [file pone.0056727.s009.pdf]

Table S2. Comparative study of the residues involved in nicotinamidase activity

**Residues**	**OiNIC**	**SpNIC**	**AbPncA**	**MtPncA**	**PhPncA**
Catalytic triad	C137	C136	C159	C138	C133
	D10	D8	D16	D8	D10
	K104	K103	K114	K96	K94
Metal binding	D54	D53	D54	D49	D52
	H56	H55	H56	H51	H54
	H72	H71	H89	H71	H71
	E65	E64	S62	H57	S60
cis-peptide bond oxyanion hole	G131	G130	G153	G132	G127
	V132	V131	I154	I133	V128
	C133	L132	A155	A134	A129
Active site forming residues	F68	F68	W86	W68	W68
	I136	I135	F158	H137	Y131
	Y107	Y106	Y123	Y103	Y103
	F15	F14	F21	F13	F15
	L22	L21	L27	L19	L21
	T105	R104	G115	G97	A95
Hydrogen bonds between main and lateral chains	T12	T11	Q18	Q10	Q12
	D14	D13	G20	D12	D14
	S108	R104	S124	S104	S104
	T141	T140	T163	T142	T137

¶OiNIC: *O. iheyensis* nicotinamidase; SpNIC: *Streptococcus pneumoniae* nicotinamidase [2]; AbPncA: *Acinetobacter baumanii* nicotinamidase [3]; MtPncA: *Mycobacterium tuberculosis* nicotinamidase [4]; PhPncA: *Pyrococcus horikoshii* nicotinamidase [5].
